# Herd health and reproductive management associated with lamb weight gain and mortality in sub-Saharan drylands—a case from Ethiopia

**DOI:** 10.1007/s11250-023-03715-z

**Published:** 2023-09-21

**Authors:** Elisabeth Genfors, Sara Lysholm, Mesfin Mekonnen Moliso, Firdawok Ayele, Barbara Wieland, Ulf Magnusson, Renée Båge

**Affiliations:** 1 Department of Clinical Sciences, Swedish University of Agricultural Sciences (SLU), Uppsala, Sweden; 2 Animal and Human Health Program, International Livestock Research Institute (ILRI), Nairobi, Kenya; 3 Animal and Human Health Program, International Livestock Research Institute (ILRI), Addis Ababa, Ethiopia; 4 Debre Berhan Agricultural Research Centre, Addis Ababa, Ethiopia; 5 Institute of Virology and Immunology, Mittelhäusern, Switzerland; 6 Department of Infectious Diseases and Pathobiology, Vetsuisse Faculty, University of Bern, Bern, Switzerland

**Keywords:** Lamb weight gain, Lamb mortality, Preventive management strategies, Sub-Saharan Africa, Ethiopia

## Abstract

Sheep are important for food and livelihood security in sub-Saharan Africa, and maximizing lamb weight gain while minimizing mortality is essential to improve production. Using the Menz sheep breeding villages of Amhara region in Ethiopia as a case study, the weight gain and mortality rate of 208 lambs were monitored during their first 5 months of life. The study was conducted in intervention and control villages, where the intervention villages were part of community-based breeding programmes and had participated in various projects aiming to improve sheep production and management. Multivariable linear regression analysis was conducted to detect associations between weight gain from birth to 1 month, and birth to 5 months, and different lamb and ewe characteristics, farmer education, application of management routines, and presence of village level sheep management interventions. In general, lambs from intervention villages, without certain signs of diseases, whose mothers were 2 years or older, had a body condition score of more than 2 on a 5-point scale, and who originated from flocks where disease prevention strategies had been implemented, had gained more weight. Overall lamb mortality was 6.8% with most deaths occurring before 1 month of age. This study highlights that health interventions in ewes improve lamb survival and weight gain and that the care of lambs during the first month of life is crucial for overall herd productivity.

## Introduction

Sheep husbandry is essential for the livelihood of many smallholder farming households in sub-Saharan Africa, including Ethiopia (Fentie et al. 2016). Sheep are not only used as a source of income or live animal savings, they are also kept for their high-quality protein in milk and meat, as a source of manure for fertilizing crop fields, for risk-diversification and for their wool and skin. The increasing demand for such products is not met due to insufficient output from the small ruminant production. One of the major constraints in the production is suboptimal animal health, often due to poor animal management (Armson et al. 2020; Hassen and Tesfaye 2014; Marshall et al. 2019).

Preventive herd health management aims to improve sheep health, fertility and performance by proper nutrition and management, good biosecurity and sanitary conditions, monitoring and early disease diagnosis, good record keeping, and strategic decisions about genetic make-up, breeding, and herd dynamics. That will in turn result in reduced disease occurrence, including subclinical diseases (Nuvey et al. 2022). Combatting subclinical disease, regardless of origin (infections, feed deficiencies, etc.), is a key factor for improved animal performance and wellbeing and enhanced production. Unfortunately, subclinical disease will often go undetected for long periods of time, which in turn might result in extensive production losses (Sargison 2020; West et al. 2009). Disease preventive management strategies increase the farmer’s profit through the improvement of animal health and welfare by the efficient utilization of natural resources (Roger 2008; Rojas-Downing et al. 2017).

Improved lamb survival and weight gain has been positively correlated to preventive herd health management, as such management improves the health of the ewe health throughout her entire reproductive cycle. Examples of such management practices are supplementary feeding of ewes in poor condition before mating and to all ewes during pregnancy and lactation, planned mating to prevent unnecessary strenuous environmental conditions at birth, proper housing with good hygiene at lambing, and monitoring and support of weak lambs (Gascoigne and Lovatt 2015; Scott 2015).

With > 40 million sheep, Ethiopia has the largest stock of small ruminants in Africa (CSA 2020). The Amhara region in the Ethiopian highlands is a densely populated area with a high ratio of small ruminants per household—despite limited access to animal feed and land area, sheep are one of the main sources of livelihood (Gizaw 2010; Lakew et al. 2021; Tilahun and Schmidt 2012). The current study was conducted in intervention and control villages in the Menz area of the Amhara region, where the intervention villages were involved in community-based breeding programmes and had participated in various projects aiming to improve sheep production in the area. These villages serve as a model for similar environments, to evaluate lamb weight gain and mortality under the conditions that characterises the area, but are also found in other parts of the African continent and elsewhere. The study aimed to monitor lamb weight gain and mortality, as well as identify associations between weight gain and selected predictor variables, including—but not limited to—ewe age, ewe health, lamb health, and presence of a number of management practices.

## Materials and methods

### Study area and study group

**Fig. 1:**
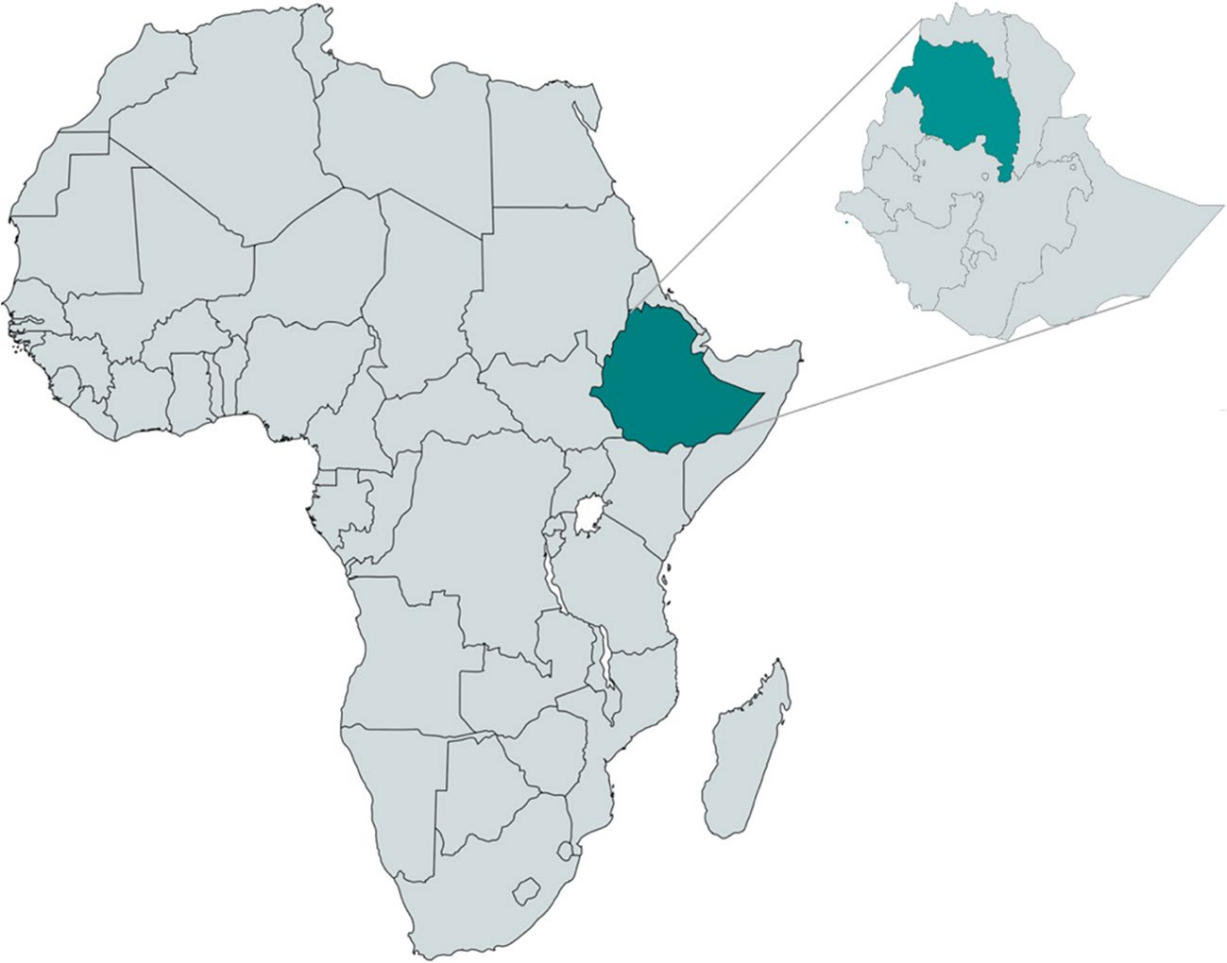
Left: Map of the African continent, with Ethiopia market in dark green. Right: Map of Ethiopia with the surveyed Amhara region marked in dark green. Source: https://mapchart.net accessed 24 November 2022. License: https://creativecommons.org/licenses/by-sa/4-0/ accessed 24 November 2022

This longitudinal study was designed to detect associations between potential predictor variables and lamb weight gain and survival in Menz area, Amhara region, Ethiopia (Fig. 1). This subalpine highland area is characterized by tepid to cool temperatures, erratic rainfall, and high occurrence of frost, making crop production challenging and thereby increasing the importance of livestock, especially sheep, for livelihood security (Gizaw et al. 2013; Haile et al. 2013). The year is subdivided into four seasons with the primary rainy season stretching from June to August, followed by the harvest season from September to November, the dry season from December to February, and the sporadic secondary rainy season from March to May (Mekonen and Berlie 2020). Mean annual rainfall in the Amhara region has been estimated to 980 mm, the majority of which falls during the wettest months (July and August), while rainfall is limited in the dry season (Abebe et al. 2020; Alemu and Bawoke 2020). Mean monthly temperatures range from approximately 5 to 18 °C, with the coldest temperatures occurring in the dry season and the hottest in May and June (Abebe et al. 2020; Mekonen and Berlie 2020).

The target population has been described previously in Genfors et al. (2023). In brief, it consisted of new-born lambs (< 7 days of age) of the Menz breed, a local short-fat-tailed breed with adult weights ranging from approximately 22 to 45 kg in rams and 20–30 kg in ewes (Getachew 2008). The sheep were kept in flocks of 5–92 (average 21, median 18) heads, by rural traditional smallholder sheep farmers residing in intervention and control villages. Intervention villages were those that had participated in previous research and capacity-building projects conducted by the CGIAR Research Program on Livestock (CRP livestock) or affiliated organizations with the aim to improve sheep management and farming in the village. Examples of such activities include vaccinations, disease surveillance, and discussion groups. The control villages were not involved in the breeding programmes and had not seen yet other interventions. Most of the sheep’s caloric and nutritional intake was obtained through grazing on communal grazing grounds within or in close proximity to the village.

### Study design

Data was collected over an 8-month period, from September 2019 to May 2020, with the aim to monitor the lambs during their first 5 months of life with focus on their survival and weight gain. Information about sheep flocks with new-born lambs was collected via local animal health workers with established contacts with the farmers. The initial visit to all sheep flocks was conducted by a research group together with the above-mentioned local animal health workers. The visit incorporated an interview with the farmer as well as an examination of the new-born lamb(s) and its’ ewe. In the event where there were more farmers with new-born lambs than the research team had time to interview in 1 day, the farmers with the oldest lambs were visited first. All examinations were conducted by the same trained veterinarian.

### Initial clinical examination of the ewes

The initial examination followed a protocol, which included registering the ewe’s ID (number if tagged, coat description if not), number of previous litters, and age. If a farmer was unsure of the age, an estimate was given, and if age was unknown, an estimate was made based on teeth status (Casburn 2016; Ridler and West 2010). Thereafter, clinical assessments of the ewes’ nutritional and health status were made and recorded by a veterinarian (Table 1).

**Table 1:** A description of how the clinical assessments of ewes and lambs were recorded during the first herd visit. Findings were noted either as normal or as deviating from normal — if deviating, a short description of observed signs of disease was made

Ewe			Lamb		
*Object of examination*	*Recorded as normal*	*Recorded as deviating from normal*	*Object of examination*	*Recorded as normal*	*Recorded as deviating from normal*
General condition	Bright, alert, lymph nodes within normal limits	Enlarged lymph nodes	General condition	Bright, alert	Stiff posture/walk, ectoparasites
Body condition score (BCS)—assessed according to Russel (1984) and Morgan-Davies et al. (2007)	BCS 2.5–3.5 on a 1–5 scale	BCS < 2.5 or BCS > 3.5 on a 1–5 scale	Joints	Non-swollen, non-tender	Traumatic injury
Fleece cleanliness	0 (clean)	> 0–3 (1 = < 25 %, 2 = 25–75%, 3 = > 75% soiled fleece)	Mouth	Intact, pink mucosal membranes	Mucosal lesions
Oral cavity	Intact, pink mucosal membranes, full set of teeth	Blisters, worn teeth, missing teeth	Eyes	Alert, no discharge, no signs of infection	Eye discharge, conjunctivitis, corneal ulcers
Eyes	Alert, no discharge	Eye discharge, impaired vision	Muzzle	No discharge, intact skin and mucosal membranes	Nasal discharge, blisters
Muzzle	No discharge, intact skin, no signs of respiratory disease	Nasal discharge, coughing, blisters, increased breathing sounds	Respiration	No signs of respiratory disease	Coughing, strenuous breathing, sneezing
Gastrointestinal tract	No signs of diarrhoea or bloating	Diarrhoea	Gastrointestinal tract	No signs of diarrhoea or bloating	Diarrhoea
Udder	Symmetrical udder, filled with milk without signs of mastitis	Empty udder, very hard udder	Umbilical cord	Non-swollen, no bleeding	Swollen umbilical cord
Milk	White/slightly yellow, homogenous milk	-	Congenital malformations	No malformations	-
Genitalia	Intact, pink mucosal membranes, no vaginal discharge	Swollen vulva, vaginal discharge			

### Clinical examination of the lambs

At the initial visit, the lambs were given an ear tag ID, unless it had already been provided by the owner, then days of age, number of siblings and weight was registered. The weight was obtained by placing the lambs in a large bag which was then hung on a scale, and the lamb’s weight was obtained by subtracting the bag’s weight from the combined weight. All lambs were then clinically examined by a veterinarian and all clinical signs recorded (Table 1). The lambs were thereafter visited once a month to examine weight and mortality. These visits were made by local animal health workers who were trained during the initial visit, and had received the necessary equipment and protocols.

### Questionnaire interview

The scoring of the responses in the questionnaire interview has been described previously in Genfors, Magnusson, Moliso, Wieland, König, Hallenberg and Båge (2023). In brief, the responses were scored using a scoring template developed by the research team. To ensure its accuracy and regional relevance, the template was verified by external sheep experts with knowledge of sheep production in Ethiopia. A high combined management score indicated high adoption of routines deemed favourable, while a low score indicated poor adoption of the same. In addition, for a more pinpointed analysis of the data, the different management routines composing the combined score were subdivided into categories, generating subscores: one for each category. The categories were divided as follows:

1. Disease prevention: Vaccinations, usage of anthelmintic drugs, restriction of flock contact with animals from other herds and actions taken to prevent lamb mortalities.
2. Mating: Mating planning and ram usage.
3. Gestation: Pregnancy control and management of pregnant ewes, including feeding routines
4. Lambing: Lambing monitoring and assistance, lambing hygiene (shearing, hand washing, usage of personal protective equipment, cleaning birthing environment), removal of placenta, and initial care of new-born lamb (clearing of airways and drying).
5. Lambs’ first week of life: Colostrum routines and lamb supervision.
6. Second week to 5 months of life: Routines concerning feeding, deworming, vaccinations, weight monitoring etc.

In addition, the education level of the farmer was included to evaluate the relationship between lamb/sheep/flock health and education. The different education levels were grouped into the following categories: no education, non-governmental or unfinished primary education, or completed primary or higher education. Non-governmental education typically signified farmers who had obtained literacy in another educational system under previous regimes, or who had been educated at church.

### Statistical analysis

**Fig. 2:**
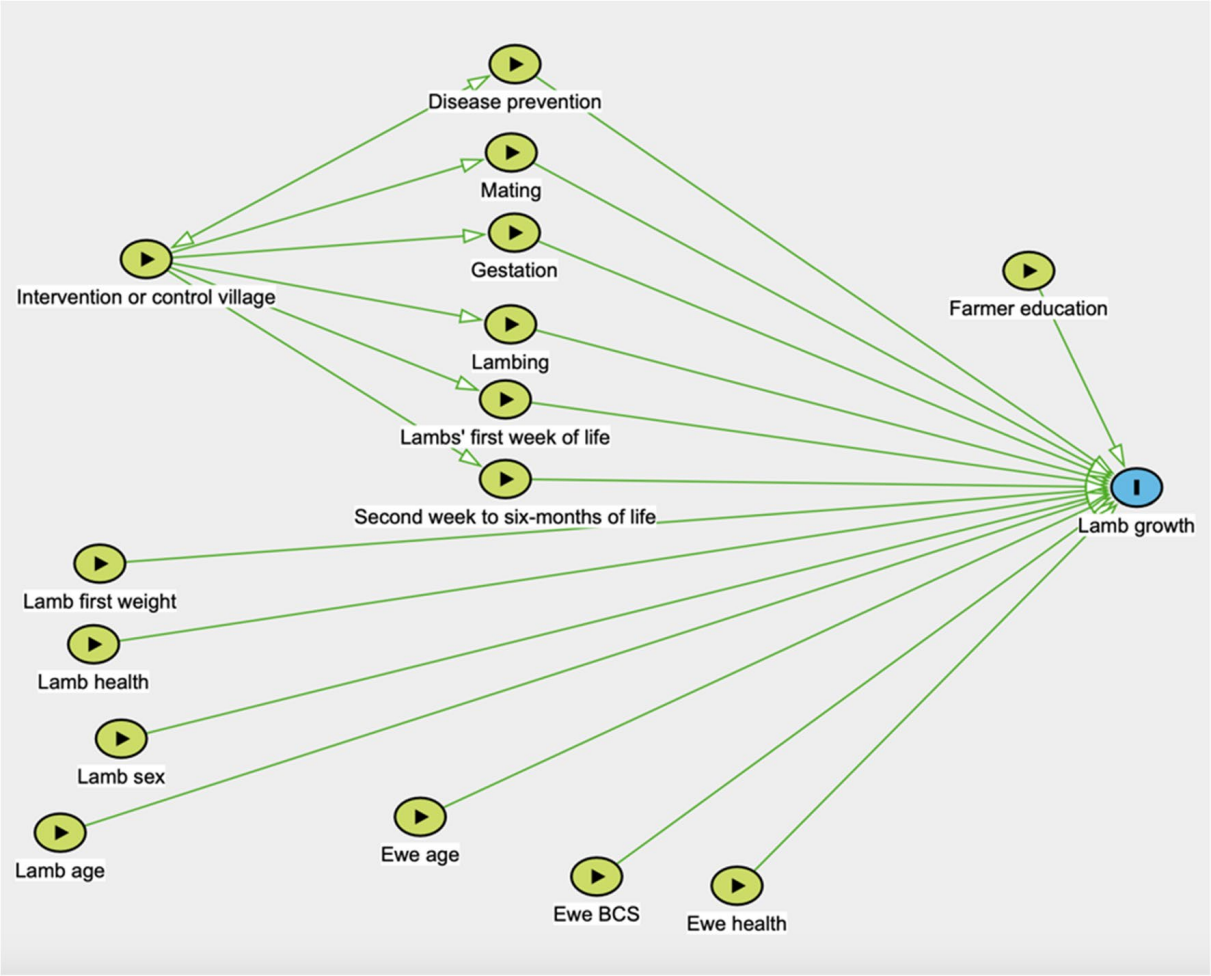
Directed acyclic graph illustrating variables that potentially were associated with lamb weight gain in Amhara region, Ethiopia. Source: http://www.dagitty.net

All data was entered into Excel 16.67 (Microsoft, USA) in preparation for the statistical analysis and to produce plots illustrating the data. Associations between lamb weight gain and potential predictor variables were identified with multilevel mixed-effects linear regression models using Stata IC 16/1 (StataCorp LLC, USA). The analysis was guided by directed acyclic graphs (Fig. 2), which identified whether the farmer resided in an intervention or a control village as a confounder, and this variable was therefore retained in all steps of the analysis. Two rounds of analysis were performed with different outcome variables, namely the lambs’ weight increase from the first to second weighing at 1 month of age, and from the first to last weighing at 5 months of age. Included predictor variables related to the lamb were weight at first visit, sex, and presence of clinical signs of disease at the initial visit. Predictor variables related to the ewe were age in years, BCS, and presence of clinical signs of disease and fleece cleanliness at the initial visit. In addition, whether the herd was located in a control or an intervention village, farmer education level, and scoring results from the questionnaire interviews were included. Litter size was not included as the study population only contained two set of twins and the rest were singletons. Random variables included in the analysis were village and household.

Initially, all variables were entered into the model and the variable with the highest conjoined *p*-value using the Wald test was removed in a stepwise backward elimination procedure, which continued until only variables with a *p*-value < 0.05 remained. Multicollinearity was tested for in all models with variance inflation factor (VIF) and a cut-off value of 10 was used. Confounding was controlled for in each step, and a variable was determined to be a confounder if it affected the coefficient of other variables with 20% or more. However, no confounder was identified. The best fitting model was selected using Akaike information criterion (AIC).

Also, univariable statistical analysis in the form of Fisher’s exact test and univariable linear and logistic regression was performed to detect associations between lamb mortality and potential predictor variables. Due to the low lamb mortality in the study and low number of predictor variables associated with lamb mortality with *p*-values < 0.25 in univariable analyses, multivariable analysis was not performed. Scoring results from the questionnaire interviews were included also in this analysis of lamb mortality, however, as opposed to in the analysis of lamb weight gain, the combined score was analysed here for simplicity reasons.

### Ethical considerations

The data was collected in collaboration with representatives from the Ethiopian veterinary system. Prior to data collection, informed written consent was acquired from each participant. The study has received an ethical approval from the ILRI Institutional Research Ethics Committee (ILRI-IREC2019-41).

## Results

### Characteristics of the study population

The population of lambs and ewes included in the study is described in Table 2. There were in total 208 lambs from 206 ewes in 143 flocks enrolled in the study, originating from seven different villages, whereof four were control and three were intervention villages. The number of surveyed lambs per household and village ranged from 1 to 6, and 23 to 46, respectively, while the number of households per village ranged from 18 to 29. Only two ewes had given birth to twins. Lamb age at the initial visit ranged from 0 to 7 days, with a median of 5 days. Almost 38% of the lambs displayed at least one clinical sign of disease at the initial visit, with the most common sign being diarrhoea (22%). Ewe age ranged from 1.5 to 8 years, with a median of 4 years. Total number of lambs per ewe summarising all parturitions ranged from 0 to 7, with a median value of 2. The ewes’ BCS ranged from 1.5 to 3, with a median value of 2. Approximately 30% of the ewes displayed clinical signs of disease at the initial visit, with signs of respiratory disease being the most common sign (10%). Around 5% of the ewes had udders that were partially or completely empty upon palpation, while 1% had udders that were judged as abnormally hard, but at visual examination, the milk of all ewes was judged as normal. Concurrent clinical signs of disease were common, with 10% of lambs and 11% of ewes displaying more than one disease sign at the initial visit.

**Table 2:** Distribution of numbers of lambs and ewes in the study population in the Menz area, Amhara region, Ethiopia

		*n*	Proportion of total (%)
*Lambs*			
Lambs	Control village	96	46.2
	Intervention village	112	53.8
	Total no.	208	100
Lamb sex	Female	107	52.2
	Male	98	47.8
	*Information missing*	3	1.4
Lambs with clinical signs of disease at initial visit	Gastrointestinal (diarrhoea)	46	22.1
	Respiratory (coughing, nasal discharge, nasal blisters)	31	14.9
	Ocular discharge	12	5.8
	Umbilical cord inflammation	11	5.3
	Other (swollen joints, signs of traumatic injury, depression, gingival blisters)	5	2.4
	Total	79	38
*Ewes*			
Ewe age class distribution	< 2 years	52	25.2
	2–4 years	77	37.4
	> 4 years	77	37.4
	Total	206	100
Ewe body condition score (1–5)	< 2	144	69.9
	> 2	60	29.0
	*Information missing*	2	0.1
Ewe fleece cleanliness (0-3)	< 1	45	21.8
	> 1–2	136	66.1
	> 2	20	9.7
	*Information missing*	5	2.4
Ewes with clinical signs of disease at initial visit	Respiratory (coughing, nasal discharge)	21	10.3
	Genitalia (genital discharge, vulval swelling)	16	7.8
	Ocular discharge	12	5.9
	Udder (empty or hard/swollen)	12	5.9
	Mouth (worn or missing teeth, gingival blisters	10	4.9
	Gastrointestinal (diarrhoea)	8	3.9
	Other (enlarged lymph nodes, depression)	7	3.4
	Total	61	29.6

### Lamb weight gain

**Fig. 3:**
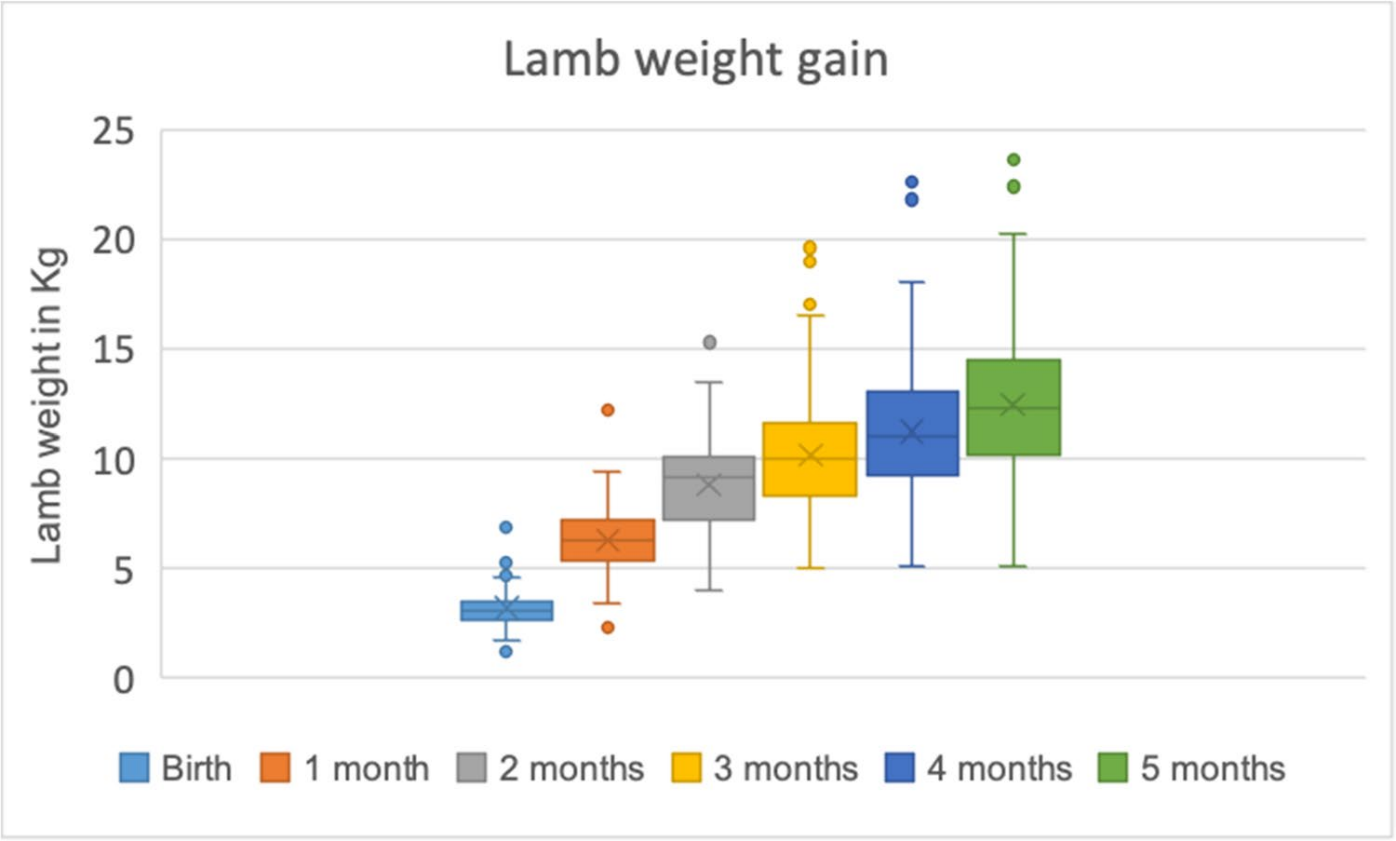
Boxplot illustrating lamb weight at different ages from birth to 5 months, in Amhara region, Ethiopia

Lamb weight gain during the study period is illustrated below in Fig. 3. At the initial visit, the weight of the lambs varied considerably as it ranged from 1.2 to 6.9 kg, with mean and median weights of 3.2 kg and 3.1 kg, respectively. After 1 month, weight gains ranged from 0.1 to 6 kg, and mean and median growth were both 3.1 kg. When comparing the first and last weight at 5 months of age, the weight gains ranged from 2.4 to 18 kg, with a mean and median growth of 9.2 kg and 9.1 kg, respectively.

### Predictor variable analysis

Predictor variables associated with lamb weight gain from 0 to 1 month of age are illustrated in Table 3, while variables associated with weight gain from 0 to 5 months of age are illustrated in Table 4.

**Table 3:** Predictor variables associated with lamb weight gain in a multi-level mixed effects linear regression, from first week to 1 month of age, in the Menz area in Ethiopia. Baseline parameter is in parenthesis in the first column. *p*-values < 0.05 are in bold. *n* = 192

Fixed-effects parameters (baseline parameter in parentheses)		Weight gain first to second weighing (0–1 month)		
		Coefficient	Coef. 95% confidence interval	*p*-value
Type of village (Control village)	Intervention village	0.89	0.38–1.41	**0.001**
Ewe age (< 2 years)	2–4 years	0.30	0.04–0.56	**0.002**
	> 4 years	0.51	0.21–0.81	**0.001**
Ewe BCS (1–5) (<2)	> 2	0.37	0.11–0.63	**0.005**
Ewe fleece cleanliness (0–3) (<1)	> 1–2	0.26	−0.01 to 0.52	0.056
	> 2	0.68	0.17–1.19	**0.009**
Ewe—abnormal udder (No)	Yes	−0.65	−1.14 to (−)0.16	**0.009**
Ewe—clinical signs from genital tract (No)	Yes	0.45	0.04–0.85	**0.030**
Lamb—ocular discharge (No)	Yes	−0.47	−0.98 to 0.04	0.072
Lamb—respiratory signs (No)	Yes	−0.35	−0.68 to (−)0.02	**0.035**
Lamb—gastrointestinal signs (No)	Yes	−0.32	−0.58 to (−)0.06	**0.016**
Lamb—umbilical cord inflammation (No)	Yes	−0.56	−1.08 to (−)0.05	**0.033**
Score—disease prevention (0–10)	11–20	0.37	0.00–0.75	0.052
Score—mating (0–10)	11–20	0.48	−0.02 to 0.98	0.062
Farmer educational level (No education)	Non-governmental or unfinished primary education	−0.51	−0.82 to (−)0.19	**0.002**
	Completed primary or higher education	−0.34	−0.72 to 0.05	0.084
Cons		2.19	1.60–2.78	< 0.001
Random-effects parameters		Estimate	Std. Err.	95% confidence interval
	Village	0.42	0.16	0.20–0.90
	Household	0.55	0.08	0.41–0.74
	Residual	0.56	0.06	0.46–0.68

**Table 4:** Predictor variables associated with lamb weight gain in a multi-level mixed effects linear regression, from first week to 5 months of age, in the Menz area in Ethiopia. Baseline parameter is in parenthesis in the first column. *p*-values < 0.05 are in bold. *n* = 179

Fixed-effects parameters (baseline parameter)		Weight gain first to last weighing (0–5 months)		
		Coefficient	Coef. 95% confidence interval	*p*-value
Type of village (Control village)	Intervention village	−0.96	−2.17 to 0.25	0.120
Ewe age (< 2 years)	2–4 years	0.93	0.31–1.55	**0.003**
	> 4 years	0.49	−0.15 to 1.14	0.136
Ewe BCS 1–5 (< 2)	>2	0.51	−0.08 to 1.09	0.092
Ewe—abnormal udder (No)	Yes	−0.82	−1.94 to 0.30	0.150
Lamb—sex (Male)	Female	−0.54	−1.02 to (−)0.05	**0.030**
Lamb—respiratory signs (No)	Yes	0.86	−1.62 to (−)0.10	**0.026**
Score—disease prevention (0–10)	11–20	0.82	0.07–1.57	**0.032**
Score—gestation (0–10)	11–20	−0.90	−1.62 to (−)0.18	**0.014**
Cons		8.94	6.52–11.4	< 0.001
Random-effects parameters		Estimate	Std. Err.	95% confidence interval
	Village	2.95	0.85	1.68–5.18
	Household	0.86	0.28	0.45–1.62
	Residual	1.43	0.15	1.16–1.76

Lamb weight gain from 0 to 1 month of age was negatively associated with presence of certain clinical signs of disease at the initial visit, namely respiratory signs (i.e. coughing and nasal discharge), gastrointestinal signs (i.e. diarrhoea), and umbilical cord inflammation. Also, the weight gain was significantly lower for lambs whose mothers had abnormal udders upon palpation (i.e. tense or partially or completely empty) at the initial visit. On the other hand, lamb weight gain was positively associated with being born by an ewe that were 2 years or older, had a BCS of more than 2 on a 5-point scale, had unclean fleece (> 2 on a 3-point scale), and had genital discharge at the initial visit. Furthermore, the analysis showed that the weight gain of lambs born in intervention villages was significantly higher compared to lambs in control villages. Also, lamb weight gain was significantly higher for farmers without education, compared to those of farmers that had obtained literacy within a non-governmental education system, or that had started but not completed primary school. Tendencies of higher weight gains were also observed in herds with higher scores for application of disease preventive strategies and favourable management routines around mating; however, these findings were not statistically significant (*p* = 0.052 and 0.062, respectively).

Lamb weight gain from 0 to 5 months of age was significantly higher for lambs whose mothers were 2–4 years old, compared to lambs born by younger ewes. Also, lamb weight gain was significantly higher for those lambs that had displayed signs of respiratory disease at the initial visit. Similarly, lamb weight gain was higher in herds that had obtained high scores for application of disease prevention strategies. However, weight gain was lower in female compared to male lambs and in lambs that originated in herds that had obtained high scores for application of favourable management strategies during the gestation period.

### Lamb mortality

**Fig. 4:**
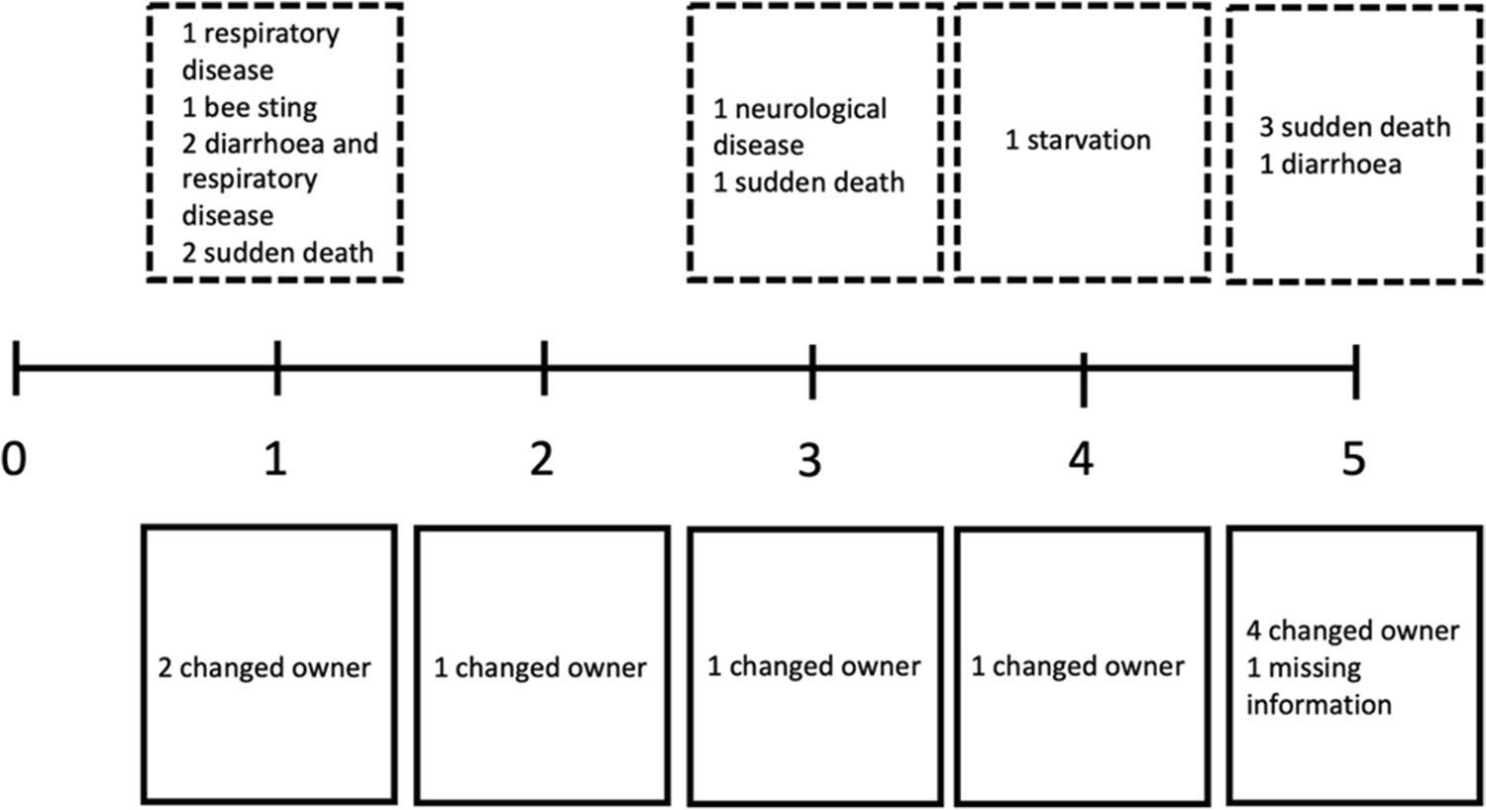
Timeline illustrating when and why the 23 lambs were lost to follow-up during the 5-month period. The dashed boxes above the timeline shows number of deaths and clinical signs manifested by the animal prior to dying, with sudden death implying that the animal died suddenly without prior clinical signs of disease. The boxes below the timeline indicate number of lambs that changed owner, or when information is missing. The numbers on the timeline indicates which follow-up month the event occurred

During the follow-up period from birth to 5 months of age, 23 lambs (12%) were lost to follow-up (Fig. 4). Of these, 13 lambs (56%) died, 9 (39%) changed owner, and for one lamb, the reason was unknown. Most of the deaths (46%) occurred before the first follow-up, while the second-most number of deaths occurred between the 4^th^ and last follow-up (31%). Causes of death in the study population included diarrhoea (*n* = 3), respiratory disease (*n* = 3), neurological disease (*n* = 1), bee sting (*n* = 1), sudden death without prior signs of disease (*n* = 5), and death of unspecified cause (*n* = 1). In univariable linear regression, an association was found between lower lamb weight at the initial visit and mortality (coefficient −0.54, *p* = 0.022). Univariable logistic regression analysis revealed that lamb mortality was associated with presence of signs of respiratory disease at the initial visit (OR 5.79, *p* = 0.003) and originating in herds that had obtained favourable combined management scores (OR 3.41, *p* = 0.038). While lamb mortality was lower in intervention than control villages (5.4% and 7.4%, respectively), the difference was not statistically significant (*p* = 0.552).

## Discussion

Sheep farming plays multiple important roles for livelihood security in sub-Saharan Africa, and for successful sheep farming, healthy lambs that grow efficiently are key components. This study demonstrated the positive effect of herd health interventions and improved managements on lamb mortality and lamb growth. Given that sheep farming is common across sub-Saharan Africa, and since similar ecosystems are found across the region, many of the findings can to varying extents be extrapolated to other countries.

### Lamb weight gain from birth to 1 month

During the first month of life, a lamb is entirely dependent on its mothers ability to produce nutrient-rich colostrum and milk in sufficient quantities, and containing adequate amounts of immunoglobulins to protect the lamb from pathogens as its immune system matures (Agenbag et al. 2021; Nowak and Poindron 2006). In this study, lambs whose mothers had udder abnormalities commonly associated with poor milk production, i.e. that were hard, or completely or partially empty, had grown significantly less during their first month of life, a finding that is in line with previous work (Grant et al. 2016). Furthermore, lambs whose mothers were 2–4 years, or older, had grown significantly more in the first month of life, potentially because older ewes are more likely to have had lambs previously and hence be experienced mothers (Aktaş et al. 2015). Also, lambs born by ewes with a BCS of more than 2 had grown significantly more, a result that is in line with previous studies (Kenyon et al. 2014). While recommendations vary for different breeds, the BCS of ewes should ideally be around 2.5–3 at lambing and early lactation, as individuals with low BCS are less able to produce milk of sufficient nutritional value and quantity to optimize lamb weight gain (Kenyon et al. 2014). Surprisingly, the study found that lambs whose mothers’ fleece was unclean (> 2 on a 3-point scale) had grown significantly more than lambs born by mothers with clean or mildly contaminated fleece (< 1 on a 3-point scale). Unclean fleece can be used as a proxy for presence of certain clinical signs of disease (i.e. diarrhoea), or for residing in unhygienic environments where disease pressure typically is higher (Kopper et al. 2014), which potentially could result in reduced lamb weight gain (Lima et al. 2020). As the opposite was seen here, the finding could indicate that fleece cleanliness is a suboptimal indicator for disease pressure for ewes and lambs that are kept in similar environments and production systems as the ones that were studied here, at least for diseases that impact on lamb weight gain. Also, the study found that lambs’ whose mothers had vaginal discharge at the initial visit had grown significantly more during their first month of life. While this finding might seem counterintuitive, it is considered normal for an ewe to have vaginal discharge in the first week after birth. These ewes may have had a quicker and more efficient emptying and involution of their uteruses than other ewes, possibly due to for example a well-functioning immune system and a good nutritional status (Scott 2015).

The current study also found that lambs that displayed either respiratory disease, gastrointestinal disease, or umbilical cord inflammation, had grown significantly less during their first month of life, which is in line with previous research (Lima et al. 2020). Lambs that are in a weakened state due to disease may struggle to find their mothers’ udder and to suckle, hence becoming even more weak from the resulting caloric and nutritional insufficiency. These lambs not only risk ending up in a downward spiral of progressively increasing weakness and feed deficiency (Scott 2015), but their immune systems are also less able to withstand diseases, further contributing to the lambs’ weakened state (Agenbag et al. 2021; Nowak and Poindron 2006). However, 11% of the lambs and 10% of the ewes displayed multiple clinical signs concurrently and that the potential impact of co-morbidities was not considered in this study. Therefore, observed associations between clinical signs and lamb weight gain should be interpreted cautiously.

Fortunately, in many cases, these negative circles can be broken with good management (Scott 2015). The current study found a positive effect of village level animal health and sheep management interventions. The surveyed households in the intervention villages had participated in research and capacity-building projects aiming to enhance sheep management and farming, which is likely to improve lamb weight gain (Genfors et al. 2023). In addition, lambs from herds where the farmer had obtained favourable scores in the questionnaire interview with regard to their disease prevention routines (more specifically vaccinations strategies, anthelmintic usage, and contact restrictions with sheep from other herds) tended to have grown more than lambs whose owners obtained lower scores; however, this finding was not statistically significant (*p* = 0.052). Similarly, the study findings indicated that lambs raised by farmers with good mating routines scores (i.e. mating planning and ram usage) had grown more than lambs whose owners obtained poorer scores, but this finding was not statistically significant (*p* = 0.062). Lastly, the analysis found that lambs kept by uneducated farmers had grown more in the first month of life compared to lambs kept by farmers who had obtained higher education. However, the difference was only statistically significant to farmers who either had obtained basic non-governmental schooling to obtain literacy, or who had started, but not completed, primary school. While it is possible that this result is a confounder, the impact on farmer education on lamb weight gain should be further explored in future studies.

### Lamb weight gain from birth to 5 months

Analysis was also made to identify associations between various predictor variables and lamb weight gain from birth to 5 months of age. It was found that lambs born by mothers in the age range 2–4 years had grown significantly more than lambs from younger ewes, which is consistent with findings in the birth to 1 month analysis. Furthermore, similar to the previous analysis, lambs born by ewes with a BCS of more than 2 tended to have grown more during the period than lambs of thinner ewes, but this finding was not statistically significant (*p* = 0.092). Also, the study detected that lambs kept by farmers who had obtained higher scores in the questionnaire interview with regard to disease prevention routines, had grown significantly more than lambs from farms that had obtained lower scores. While indications for the same was seen also in the analysis of lamb weight gain from birth to 1 month, the finding was not significant, indicating that application of disease preventive strategies is especially important slightly for older lambs. Also, contradictory to the findings in the previous analysis, lambs with signs of respiratory disease at birth had grown significantly more during the 5-month period than lambs without respiratory signs. This could indicate that while the weight gain of lambs that develop e.g. nasal discharge and coughing early on in life might be stunted initially, they can recover and later even surpass lambs that were apparently healthy after birth. Again, as co-morbidities were not accounted for in the analysis, the associations between clinical signs and lamb weight gain should be interpreted with caution.

In addition, the analysis yielded that female lambs had grown significantly less than male lambs during the study period, which is in line with findings in previous studies (Okeudo and Moss 2008; Simeonov et al. 2014). Surprisingly however, households that had obtained high scores on gestation routines had significantly lower lamb weight gain than households with lower scores. In a previous study, favourable gestational routines have been significantly associated with low lamb mortality rates (Genfors et al. 2023). While lamb mortality rate and weight gain are different parameters, they are to a large extent influenced by similar internal and external factors, such as disease occurrence, food access, and similar. It is hence likely that this association is a confounder for other variables.

### Lamb mortality

In previous studies in Ethiopia, mortality rates of up to 50% or more have been reported (Ayele and Urge 2018; Fentie et al. 2016; Mukasa-Mugerwa et al. 2000; Mukasa-Mugerwa et al. 1994). In the current study, 13 lambs (6.3%) died during the follow-up period. In previous work, disease and malnutrition have been identified as the most common causes of lamb mortality in Ethiopia (Fentie et al. 2016). Since malnutrition is linked to feed availability, year by year variability is normal and depends on climatic events. In the current study, six of the lambs (46%) died suddenly without appearing clinically ill prior to death, while five (38%) had displayed respiratory signs, diarrhoea, or neurological signs shortly before dying. While starvation was only stated as the cause of death for one lamb, insufficient caloric and nutritional intake can also result in increased susceptibility and vulnerability to diseases, thereby indirectly contributing to mortality (Asín et al. 2021). About half of the lambs that died did so in the first month of life, which is in line with findings in previous research (Fentie et al. 2016). Also, a considerable proportion of the lambs that died did so between months 4 and 5. At this time-point, they are completely weaned and hence entirely dependent on access to good grazing material. The fifth follow-up occurred in mid-February to late April, which in the Amhara region is towards the end and just after the dry season (Mekonen and Berlie 2020). A potential reason for the increased mortality during this time is hence poor water and feed availability.

In univariable linear regression analysis, it was found that the lambs that died had significantly lower weights at the initial visit, compared to the lambs that survived the whole study period, a finding that is in line with previous work (Christley et al. 2003; Flinn et al. 2020). Furthermore, the analysis showed that lambs that had respiratory signs of disease at the initial visit were significantly more likely to die compared to lambs without respiratory signs, corresponding to findings in previous studies in Ethiopia (Fentie et al. 2016; Mukasa-Mugerwa et al. 2000). Surprisingly, lambs that originated from herds with high combined management scores were found to be significantly more likely to die during the follow-up period than lambs from herds with lower scores. This is the opposite of previous findings in a study conducted by the authors in the same area and with the same respondents, but applying a multivariate analysis of retrospective data on lamb mortality (Genfors et al. 2023). However, in the current study, multivariable analysis was not conducted due to a low number of mortalities in the study population and the findings should therefore be interpreted cautiously.

### Study significance

Enhancing lamb weight gain while reducing lamb mortality is crucial for any sheep enterprise to be successful. In this study, several variables that are associated with lamb weight gain were discovered, and valuable information on lamb mortality was collected. The generated knowledge can be used to improve sheep production in general, and lamb management in particular, for example, by providing farmers with the necessary knowledge and resources to ensure good nutritional status and health in lambs and ewes. Furthermore, by educating farmers on the importance of providing extra good care to lambs during the critical first month of their lives, flock output can be increased. While the current study was performed in the Amhara region, Ethiopia, much of the generated knowledge can to varying extents be extrapolated and utilized in similar production systems and environmental contexts across sub-Saharan Africa.

### Study limitations

In spite of the interesting findings of this study, there are some limitations. Some of the effects observed were not statistically significant, indicating that the effects are multi-factorial. For future studies, it is thus recommended to increase the sample size and to run the study over several years to be able to account for other factors and interactions between factors and to account for seasonal and climatic weather effects. For practical reasons, clinical signs of disease in the lambs and ewes were only recorded at the initial visit, as this was the only time during the data collection process when a veterinarian could be present. Due to the impact of disease on lamb weight gain, documenting signs of disease throughout the study period would have enriched the study. The association between feeding routines and lamb weight gain and mortality was not investigated in detail, but we recommend that this aspect is studied in-depth in future studies. While udder abnormalities were recorded in twelve ewes, the milk of all lactating animals was deemed normal. However, the analysis was limited to ocular inspection, and it is therefore possible that milder abnormalities in the milk was missed. More detailed examination, e.g. in the form of bacteriological analysis, would have added interesting information to the study.

## Conclusions

This field study highlights health interventions for ewes as a mean to improve lamb survival and weight gain, and that the care of lambs in their first month of life is crucial for sheep farming in resource-poor production systems. Improved herd health would not only lead to better animal health and welfare, but can also contribute to reduce poverty, increase resilience and alleviate hunger.
